# An Unexpected Inactivation of *N*-Heterocyclic Carbene Organic Catalyst by 1-Methylcyclopropylcarbaldehyde and 2,2,2-Trifluoroacetophenone

**DOI:** 10.3389/fchem.2022.875286

**Published:** 2022-03-24

**Authors:** Yanling Chen, Jie Lv, Xuling Pan, Zhichao Jin

**Affiliations:** State Key Laboratory Breeding Base of Green Pesticide and Agricultural Bioengineering, Key Laboratory of Green Pesticide and Agricultural Bioengineering, Ministry of Education, Guizhou University, Guiyang, China

**Keywords:** catalyst inactivation, N-heterocyclic carbene, organocatalysis, 3-component reaction, multi-step cascade cyclization

## Abstract

An unprecedented inactivation process of the indanol-derived NHC catalysts bearing *N*-C_6_F_5_ groups is reported. An unexpected multi-cyclic complex product is obtained from the 3-component reaction with the 1-methylcyclopropyl-carbaldehyde, the 2,2,2-trifluoroacetophenone and the NHC catalyst. The absolute structure of the inactivation product is unambiguously assigned *via* X-ray analysis on its single crystals. The formation of the structurally complex product is rationalized through a multi-step cascade cyclization process.

## Introduction

Ever since the first report from Ukai and co-workers on the thiazolium salt promoted benzoin reaction in 1943 (Ukai et al., 1943), *N*-heterocyclic carbene (NHC) has been developed and used as robust organic catalyst for more than 70 years (Breslow, 1958; Sheehan and Hunneman, 1966; Enders et al., 1995; Rovis et al., 2002). Especially, NHC organocatalysis has seen fantastic development within the past two decades (Enders et al., 2007; Lupton et al., 2013; Glorius et al., 2014; Mahatthananchai and Bode, 2014; Nair et al., 2015; Rovis et al., 2015; Scheidt et al., 2018; Chi et al., 2020; Chi et al., 2021; Wang et al., 2017). Numerous catalytic activation modes have been established within this highly active research field with a huge number of reactions realized for quick and selective access to functional molecules with interesting synthetic or biological applications. Functional molecules such as aldehydes, carboxylic acids and their derivatives, imines, ketenes, and activated ketones can be efficiently activated by NHC organic catalysts *via* formation of (*aza*)-Breslow intermediates and go through addition reactions with various electrophiles or nucleophiles through electron-pair-transfer processes (Gu et al., 2017; Yao et al., 2019; Chen et al., 2020; Yao et al., 2020; Fu et al., 2021; Xue and Zheng, 2021). Due to the rich electron densities of the Breslow intermediates formed from the NHC catalysts and the aldehyde substrates, they can be selectively oxidized by external oxidants through single-electron-transfer (SET) processes and furnished radical reactions in both enantioselective and non-chiral fashion. Recently, a couple of carbon- and heteroatom-centered nucleophiles were found to be activated by chiral NHC catalysts *via* non-covalent interactions and smoothly participate in the enantioselective addition reactions with a diversity of electrophiles (Van Halbeek and Poppe, 1991; Enders et al., 1995; Regitz, 1996; Enders, 2003; Bastin et al., 2019). In all the NHC-catalyzed synthetic transformations we mentioned above, mechanistic studies *via* both experimental and computational methods have played critical roles in the development and innovations of the activation modes. Therefore, the observation and characterization of the critical intermediates and/or side reaction products to provide evidence for mechanistic studies are of great significance.

**FIGURE 1:**
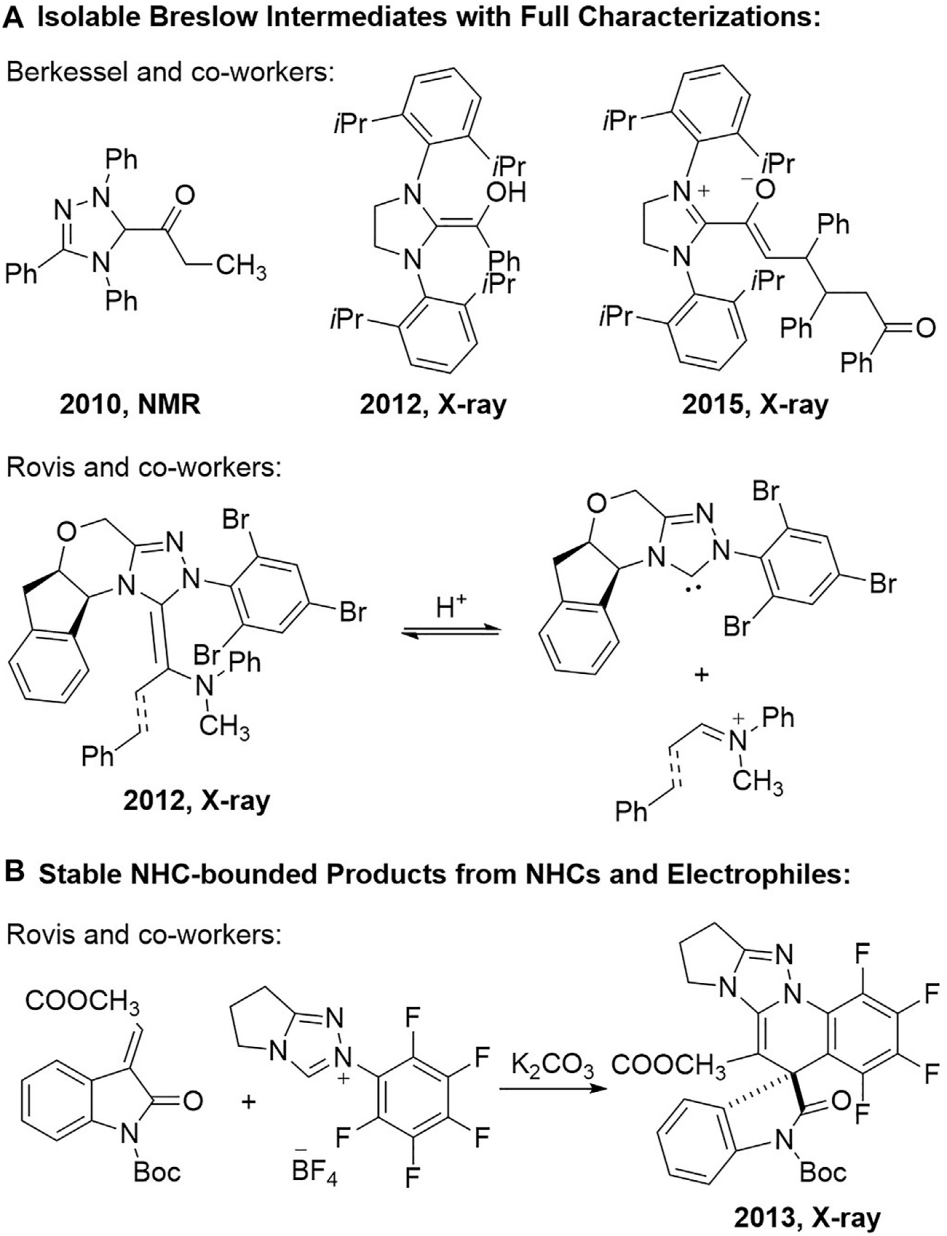
Isolation and characterization of NHC-bounded intermediates and products. **(A)** Isolable breslow intermediates with full characterizations. **(B)** Stable NHC-bounded products from NHCs and electrophiles.

Investigations into the cross-interactions between the NHC organic catalysts and the reaction substrates are one of the effective approaches for the mechanistic studies in NHC organocatalytic reactions. Continuous endeavour has been made by organic chemists towards the isolation and characterization of the most basic Breslow intermediates since it was hypothesized by Breslow in 1958 (Breslow, 1958) (Figure 1A). For example, Berkessel and co-workers reported in 2010 the full NMR spectra analysis of the ketone form of the Breslow intermediate generated from the triazolium salt-derived NHC catalyst and the propionic aldehyde (Berkessel et al., 2010). They successfully isolated the crystals of the typical Breslow intermediate from an imidazolium-typed NHC catalyst and the benzaldehyde and obtained its X-ray analytical spectrum in 2012 (Berkessel et al., 2012). An advanced reaction intermediate between the *α*,*β*-unsaturated Breslow intermediate and the chalcone substrate could also be isolated as stable crystals and their structures were unambiguously assigned *via* X-ray analysis in 2015 (Berkessel et al., 2015). The single crystals of the *aza*-analogues of the Breslow intermediate were obtained by Rovis and co-workers from a chiral indanol-derived NHC catalyst and an iminium salt in 2012 (Rovis et al., 2012). They can also apply the *aza*-Breslow intermediate analogues as the NHC catalyst precursors to promote an intramolecular Stetter reaction in enantioselective fashion.

The isolation and characterization of the NHC-bounded reaction products from the inactivation of the NHC catalysts in the reaction system can also provide significant information on the cross interactions between the NHC catalysts and the reaction substrates. Although the formation of the NHC-bounded side reaction products might be sometimes observed during the investigations of various NHC organocatalytic transformations, to the best of our knowledge, there has been very limited reports on the characterization of those NHC-bounded reaction products (Berkessel et al., 2013; Berden et al., 2021). A representative study was from Rovis and co-workers in 2013, when they reported a cascade cyclization reaction between the triazolium NHC catalyst bearing an *N*-pentafluorophenyl (*N*-C_6_F_5_) group and the isatin-derived *α*,*β*-unsaturated ester substrate (Rovis et al., 2013) (Figure 1B). The spirocyclic product was characterized *via* X-ray analysis on the product crystals.

**FIGURE 2:**
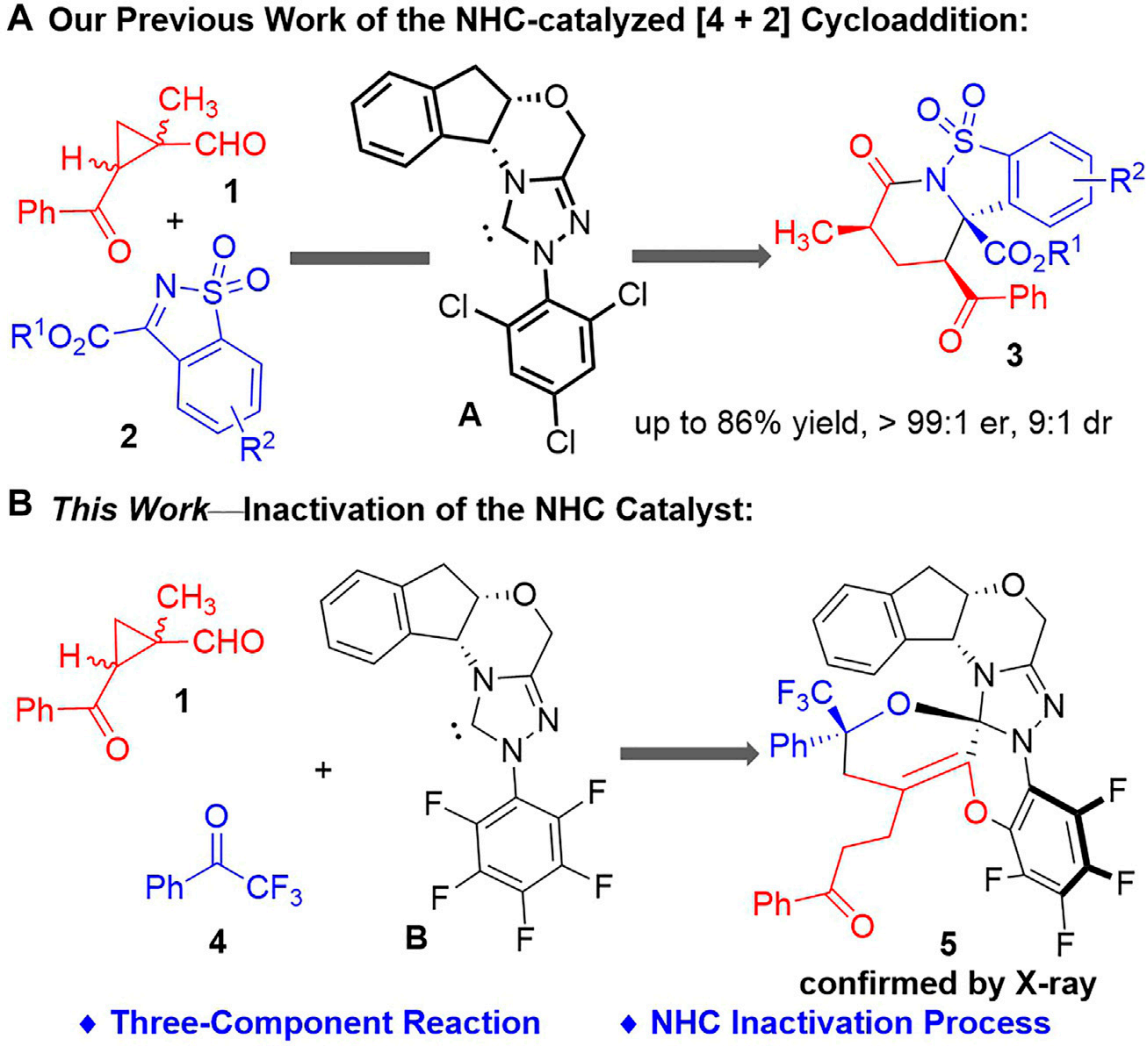
Our studies on 1-methylcyclopropylcarbaldehyde activations with NHC organic catalysts. **(A)** Our previous work of the NHC-catalyzed (4 + 2) cycloaddition. **(B)** This work—inactivation of the NHC catalyst.

We have previously reported an NHC-catalyzed asymmetric (4 + 2) cycloaddition reaction between 1-methylcyclopropyl-carbaldehyde **1** (Chi et al., 2011; Mu et al., 2020; Tong et al., 2021; Wang et al., 2021) and the cyclic sulfonimides **2** (Xu et al., 2013; Wang et al., 2020a; Wang et al., 2020b; Liu et al., 2021) to give a variety of multi-functionalized fused cyclic products **3** in moderate to good yields with generally excellent enantio- and diastereoselectivities (Lv et al., 2021) (Figure 2A). Indanol-derived NHC catalysts bearing electron-deficient *N*-substituents were found effective for this transformation. However, during the evaluations of different chiral NHC catalysts, we noticed that switching the indanol-derived NHC catalyst **A** (Rovis et al., 2012) to the NHC catalyst **B** (Enders and Balensiefer, 2004) bearing an *N*-C_6_F_5_ group resulted in a significant drop of the product yields (from 72% with **A** to 36% with **B**), with multiple unidentifiable by-products formed in low yields.

After completion of our studies on the chiral NHC-catalyzed [4 + 2] cycloaddition reactions. We continue to focus on the side products formed with the NHC catalyst **B** bearing an *N*-C_6_F_5_ group (Figure 2B). To our delight, a crystalline product could be isolated from the reaction system consisted of the 1-methylcyclopropyl-carbaldehyde **1**, the 2,2,2-trifluoroaceto-phenone **4** (Su et al., 2017) and the NHC catalyst **B**. An unexpected crystal structure of **5** was assigned by X-ray analysis, with all the three components combined within one molecule.

## Results and Discussion

Having obtained the crystal structure of the NHC-bounded compound **5**, we went on to optimize the reaction condition in order to improve the yield of the structural complex product **5** (Table 1). The raw materials of the 1-methylcyclopropyl-carbaldehyde **1**, the 2,2,2-trifluoroacetophenone **4** and the NHC catalyst **B** were initially stirred in THF at room temperature in the presence of a stoichiometric amout of Et_3_N, with the product of **5** obtained in 12% yield (Table 1, entry 1). The yield of the target product **5** could be improved when switching Et_3_N in to stronger bases such as DBU and Cs_2_CO_3_ (entries 2-3). Solvents other than THF we tested were not effective for this transformation (e.g., entries 4-6). The attempts to improve the reaction yield by adding more or less amount of basic additives were failed (entries 7-8). Further increasing the reaction temperature resulted in the formation of the target product with the same yield.

**TABLE 1:** Optimization of reaction conditions^a^. 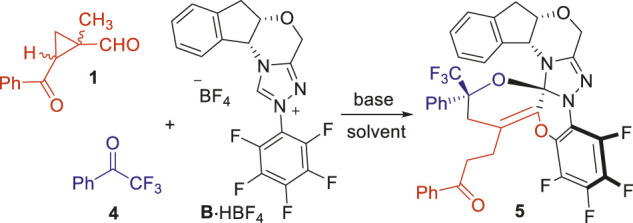

**FIGURE 3:**
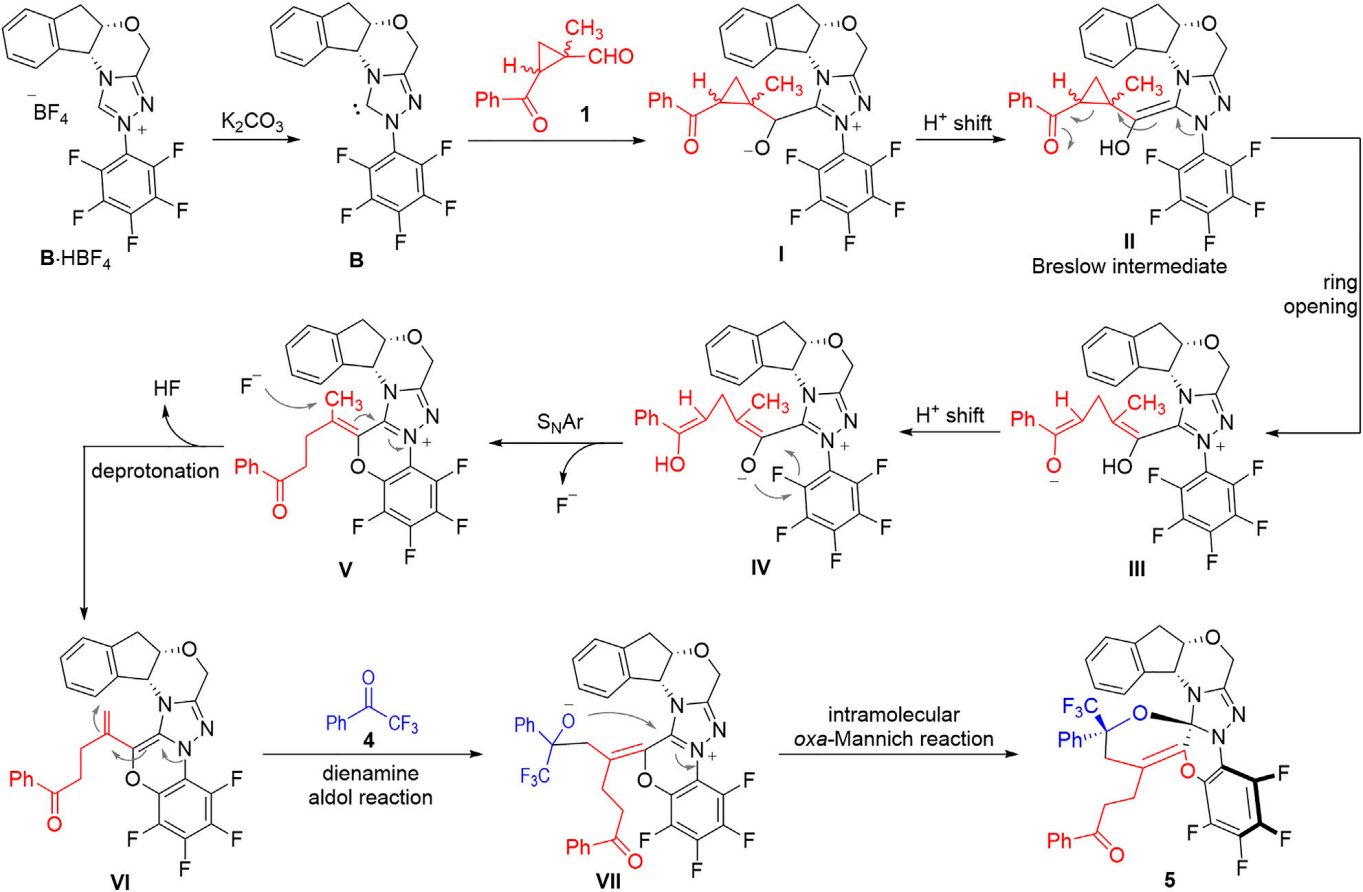
Proposed reaction mechanism for the formation of the product **5**.

The formation of the structurally complex product **5** is rationalized through a multi-step cascade cyclization process among the three components of the 1-methylcyclopropyl-carbaldehyde **1**, the 2,2,2-trifluoroacetophenone **4** and the NHC catalyst **B** (Figure 3). After deprotonation of the NHC pre-catalyst, the free NHC **B** can attack the aldehyde **1** to give the adduct **I**, which can isomerize to give the Breslow intermediate **II** *via* an intramolecular proton shift process. The Breslow intermediate **II** can go through a ring-opening process to give the zwitter ionic intermediate **III** that is in equilibrium with the intermediate **IV** through intramolecular proton transfer processes. The oxide anion of the intermediate **IV** can attack the electron-deficient pentafluorophenyl group to form a 6-membered ring *via* an intramolecular O-addition/elimination (S_N_Ar) process to give the intermediate **V**. The intermediate **V** bears an *α*,*β*-unsaturated iminium ion moiety that can easily be deprotonated by the F^−^ anion to form the dienamine intermediate **VI**. A dienamine aldol reaction between the intermediate **VI** and the 2,2,2-trifluoroacetophenone **4** gives the adduct **VII**, which can further cyclize to afford the final product **5** *via* an intramolecular *oxa*-Mannich reaction process.

It is worth noting that the formation of the structurally complex compound **5** is a highly stereospecific process. Only one diastereomer is observed in all the experiments we carried out. This is probably due to the steric match/mismatch effects provided by the chiral NHC scaffold we used in this transformation.

## Conclusion

In summary, we have disclosed an unprecedented inactivation process of NHC organic catalysts bearing *N*-C_6_F_5_ groups. A structurally complex multi-cyclic compound was obtained from the 3-component reaction of the 1-methylcyclopropyl-carbaldehyde, the 2,2,2-trifluoroacetophenone and the NHC catalyst bearing an *N*-C_6_F_5_ group. The absolute structure of the complex product was unambiguously assigned *via* X-ray analysis on its single crystals. The current study can provide novel inspections into the possible pathways that are taking place in the reactions promoted by NHC catalysts bearing *N*-C_6_F_5_ groups. Further investigations into the interactions between the NHC organic catalysts and various reaction substrates are in progress in our laboratories.

## Figures and Tables

**Table:** 

Entry	Base	Equivn. Of base	T (^o^C)	Solvent	Yield (%)^b^
1	Et_3_N	1.0	30	THF	12
2	DBU	1.0	30	THF	21
3	Cs_2_CO_3_	1.0	30	THF	34
4	Cs_2_CO_3_	1.0	30	DCM	<5
5	Cs_2_CO_3_	1.0	30	CHCl_3_	<5
6	Cs_2_CO_3_	1.0	30	MeCN	<5
7	Cs_2_CO_3_	0.8	30	THF	31
8	Cs_2_CO_3_	1.5	30	THF	27
9	Cs_2_CO_3_	1.0	50	THF	34

a Unless otherwise specified, the reactions were carried using **1** (0.05 mmol), **4** (0.05 mmol), **B**·HBF_4_ (0.05 mmol), base, 4 Å MS (50.0 mg), solvent (1.0 ml) at the given temperature for 6 h.

b Isolated yield of **5**.

## Data Availability

The raw data supporting the conclusion of this article will be made available by the authors, without undue reservation.
