# Supplementary material for: An Unexpected Inactivation of N-Heterocyclic Carbene Organic Catalyst by 1-Methylcyclopropylcarbaldehyde and 2,2,2-Trifluoroacetophenone
Source: Front Chem. 2022 Mar 24;10:875286. doi: 10.3389/fchem.2022.875286 (PMC8988059; doi:10.3389/fchem.2022.875286)
Supplement: Supplementary file 1 [file DataSheet1.pdf]

**Supporting Information**

# An Unexpected Inactivation of *N*-Heterocyclic Carbene Organic Catalyst by 1-Methylcyclopropylcarbaldehyde and 2,2,2-Trifluoroacetophenone

Yanling Chen,† Jie Lv,† Xuling Pan, and Zhichao Jin*

State Key Laboratory Breeding Base of Green Pesticide and Agricultural Bioengineering, Key Laboratory of Green Pesticide and Agricultural Bioengineering Ministry of Education, Guizhou University, Huaxi District, Guiyang 550025, China.

[†] These authors contributed equally to this work.

E-mail: zcjin@gzu.edu.cn

## table of content

I. General information ........ S2
II. Preparation of substrates ........ S3
III. General procedure for the catalytic reactions ........ S4
IV. Stereochemistry determination by X-ray crystallographic analysis. ........ S5
V. Characterization of substrates and products ........ S6
VI. $^{1}$H NMR, $^{13}$C NMR, $^{19}$F NMR spectra ........ S7

## I. General information

Commercially available materials purchased from Energy Chemical and J&K were used as received. Unless otherwise specified, all reactions were carried out under an atmosphere of Air in 4.0 mL vial. NMR spectra were measured either on a JEOL-ECX-500 (500 MHz) or on a Bruker ASCEND 400 (400 MHz) spectrometer. The chemical shift values were corrected to 7.26 ppm ($^{1}$H NMR) and 77.16 ppm ($^{13}$C NMR) for $CDCl_3$. $^{1}$H NMR splitting patterns are designated as singlet (s), double (d), triplet (t), quartet (q), doublet of doublets (dd), multiplets (m), and etc. All first-order splitting patterns were assigned on the base of the appearance of the multiplet. Splitting patterns that could not be easily interpreted are designated as multiplet (m) or broad (br). High resolution mass spectrometer analysis (HRMS) was performed on Thermo Fisher Q Exactive mass spectrometer. Optical rotations were measured on a Insmark IP-digi Polarimeter in a 1 dm cuvette. The concentration (c) is given in g/100 mL. Melting Point (MP): Melting points were measured on a Beijing Tech Instrument X-4 digital display micro melting point apparatus and are uncorrected. Analytical thin-layer chromatography (TLC) was carried out on pre-coated silica gel plate (0.2 mm thickness). Visualization was performed using a UV lamp.

## II. Preparation of substrates

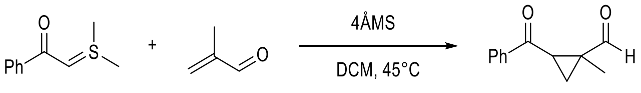


S1 S2 S3

To a solution of **S2** (5.0 mmol) in $CH_2Cl_2$ (25 mL) were added 4Å molecular sieves (0.5 g), **S1** (6.0 mmol). The mixture was refluxed for 2-6 h, and then cooled to room temperature. The crude mixture was filtered through celite and the residue was washed with $CH_2Cl_2$ (100 mL) to afford a pale yellow solution. After removal of the solvent in vacuum, the residue was purified via flash column chromatography to give the desired product **S3**.[1]

## References:

[1] a) M. F. Oswald, S. A. Raw, R. J. Taylor, *Org. Lett.* **2004**, *6*, 3997-4000; b) G. Q. Li, L. X. Dai, S. L. You, *Org. Lett.* **2009**, *11*, 1623-1625; c) H. Lv, J. Mo, X. Fang, Y. R. Chi, *Org. Lett.* **2011**, *13*, 5366-5369; d) J. Lv, J. Xu, X. Pan, Z. Jin, Y. R. Chi, *Sci. China Chem.* **2021**, *64*, 985-990.

## III. General procedure for the catalytic reactions

### General procedure for the synthesis of product 5:

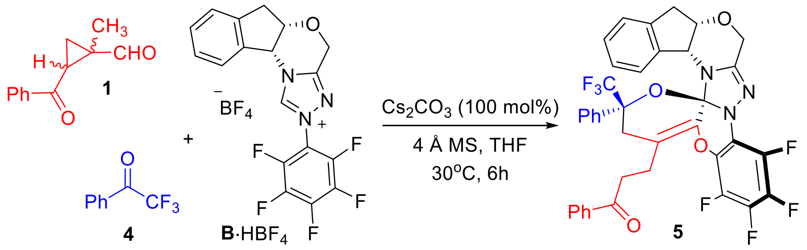


To a 4.0 mL vial equipped with a magnetic stir bar was added NHC pre-catalyst **B**.$HBF_4$ (0.05 mmol, 23.4 mg), $Cs_2CO_3$ (0.05 mmol, 16.3 mg), 4Å molecular sieves (50 mg), and freshly distilled anhydrous THF (1.0 mL) was added *via* syringe, then added substrates **1** (0.05 mmol) and substrates **4** (0.05 mmol) in glovebox. The reaction mixture was allowed to stir for 6 hours at 30 °C. After completion of the reaction, monitored by TLC plate, the mixture was concentrated under reduced pressure. The resulting crude residue was purified *via* column chromatography on silica gel (20:1 to 10:1 petroleum ether / EtOAc) to afford the desired product **5**.

## IV. Stereochemistry determination by X-ray crystallographic analysis.

A colorless block crystal of **5** was obtained by vaporization of its EtOH / $CH_2Cl_2$ / petroleum ether solution.

CCDC **2133236** contain the supplementary X-ray crystallographic data of **5**. These data can be obtained free of charge from The Cambridge Crystallographic Data Centre via www.ccdc.cam.ac.uk/data request/cif.

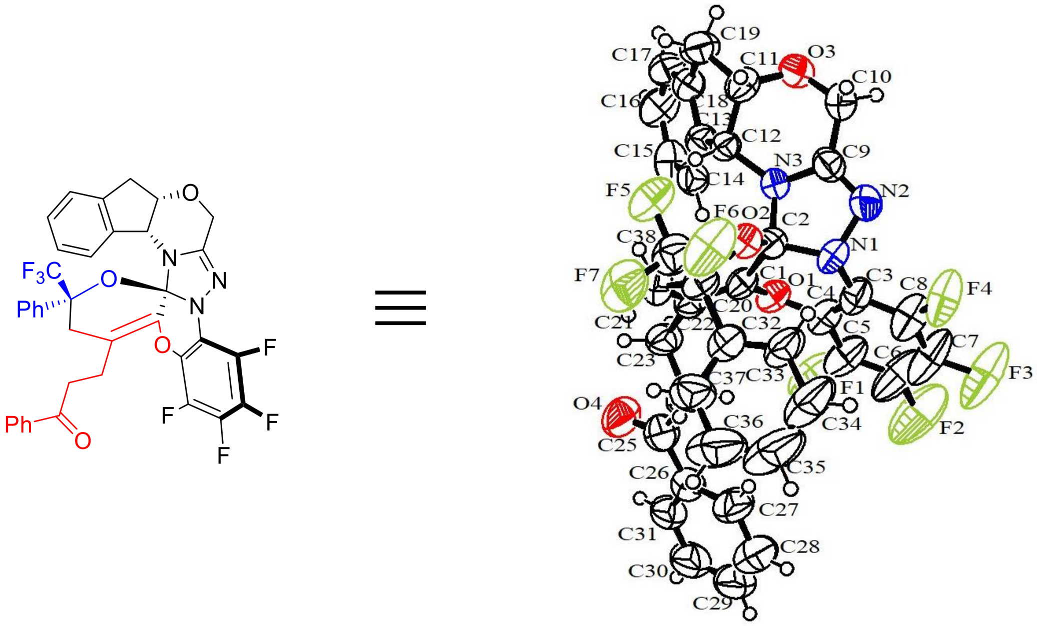

## V. Characterization of substrates and products

**Compound 5:**

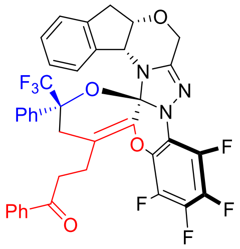


White solid, 34% yield, 12.3 mg; m.p. 123-125 $^{o}$C;

**$[\alpha]^{12}$D** = -65.3 (*c* = 1.0 in $CHCl_3$);

**$^{1}$H NMR** (400 MHz, $CDCl_3$) δ 7.85 – 7.80 (m, 2H), 7.79 – 7.74 (m, 2H), 7.61 – 7.56 (m, 1H), 7.48 – 7.42 (m, 2H), 7.32 – 7.26 (m, 4H), 7.26 – 7.17 (m, 3H), 5.06 (d, *J* = 3.8 Hz, 1H), 4.66 (d, *J* = 1.9 Hz, 2H), 4.47 (t, *J* = 4.1 Hz, 1H), 3.27 (dd, *J* = 16.7, 4.4 Hz, 1H), 3.23 – 3.11 (m, 3H), 3.10 – 3.04 (m, 2H), 2.93 – 2.77 (m, 2H).

**$^{13}$C NMR** (101 MHz, $CDCl_3$) δ 197.9, 152.3, 140.0, 140.0, 138.3, 136.2, 133.6, 133.3, 129.4, 128.7, 128.4, 128.4, 128.2, 128.0, 127.4, 127.1, 125.5, 124.0, 123.9 (q, *J* = 282.1 Hz), 120.2, 120.0, 97.3, 81.1 (q, *J* = 29.1 Hz), 78.7, 77.2, 61.6, 57.9, 37.4, 35.5, 30.7, 29.7, 24.7.

**$^{19}$F NMR** (377 MHz, $CDCl_3$) δ -80.0, -148.9 (dd, *J* = 21.5, 7.9 Hz), -161.3 (dd, *J* = 21.1, 7.9 Hz), -162.1 (t, *J* = 21.5 Hz), -164.2 (t, *J* = 21.4 Hz).

**HRMS** (ESI, m/z) calcd. for $C_{38}H_{26}F_7N_3O_4H^+$: 722.1884, found: 722.1861.

# VI. $^{1}$H NMR, $^{13}$C NMR, and $^{19}$F NMR spectra

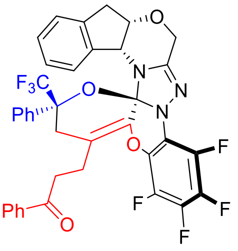


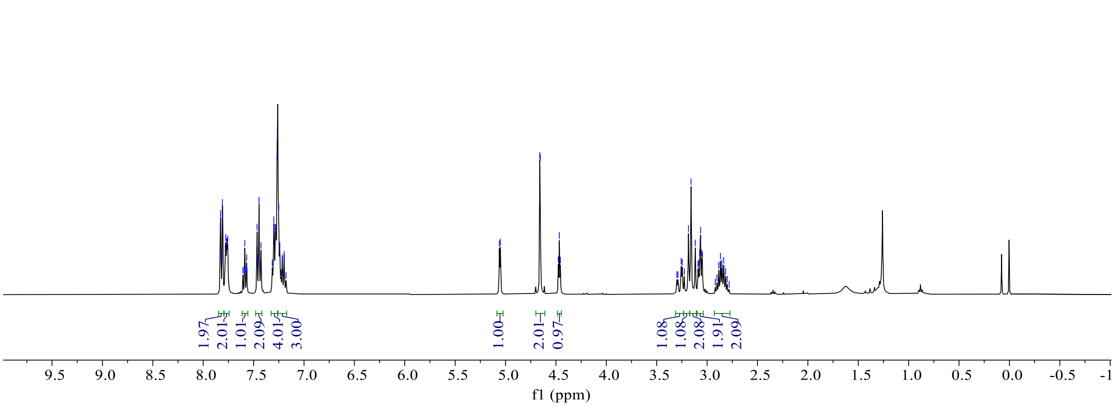


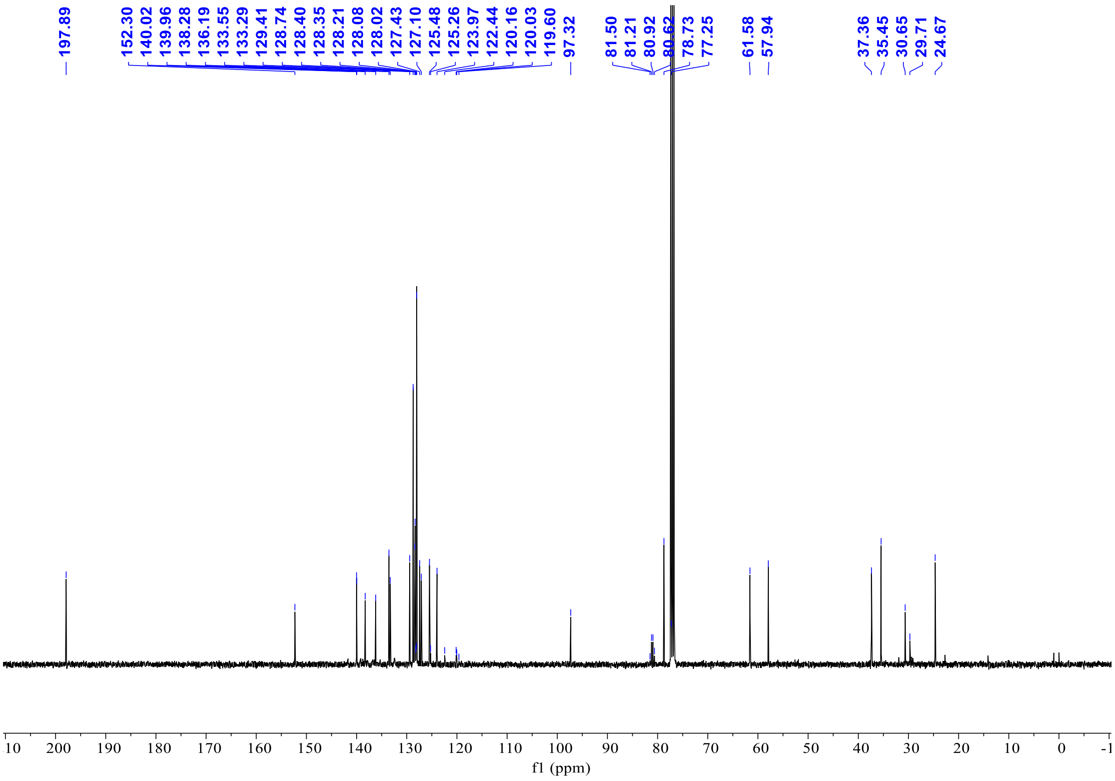

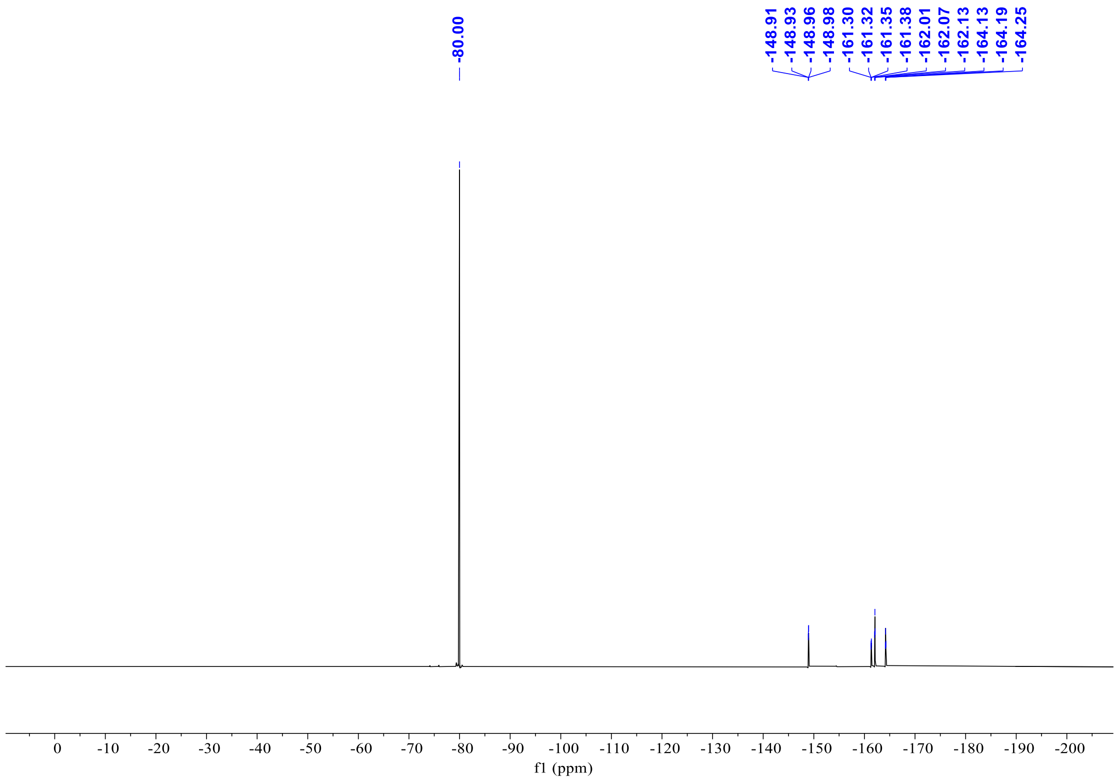
-80.00
-148.91
-148.93
-148.96
-148.98
-161.30
-161.32
-161.35
-161.38
-162.01
-162.07
-162.13
-164.13
-164.19
-164.25
0 -10 -20 -30 -40 -50 -60 -70 -80 -90 -100 -110 -120 -130 -140 -150 -160 -170 -180 -190 -200
f1 (ppm)
